# Prognostic value of total bilirubin level for patients suffering from coronary heart disease undergoing percutaneous coronary intervention: a meta-analysis

**DOI:** 10.3389/fcvm.2026.1783537

**Published:** 2026-07-30

**Authors:** Zhouxin Wu, Jinchuang Li, Jing Sun

**Affiliations:** 1 First School of Clinical Medicine, Heilongjiang University of Chinese Medicine, Harbin, Heilongjiang, China; 2 Department of Cardiovascular Medicine, First Affiliated Hospital of Heilongjiang University of Traditional Chinese Medicine, Harbin, Heilongjiang, China

**Keywords:** all-cause mortality, coronary heart disease, CV death, MACE, meta-analysis, PCI, total bilirubin

## Abstract

**Objective:**

The objective of this study is to explore the prognostic value of serum total bilirubin for major adverse cardiovascular events (MACE), cardiovascular death (CV death), and all-cause mortality in coronary heart disease (CHD) patients after percutaneous coronary intervention (PCI).

**Methods:**

Databases (PubMed, Cochrane Library, Embase, Web of Science) were searched up to May 2026). Studies were screened via PICOS. Two researchers independently extracted data and assessed study quality using Newcastle-Ottawa Scale. Meta-analysis was performed using STATA 16.0. Subgroup analysis was performed to explore the source of heterogeneity, and Egger's test was conducted to examine publication bias.

**Results:**

In total, 17 observational studies involving 27,580 patients with CHD undergoing PCI were included. Methodological quality was generally high (NOS score ≥7). The primary analyses were performed with *a priori* stratification by follow-up setting. In the long-term follow-up subgroup (12 studies), although a high total bilirubin level was significantly associated with a lowered risk of MACEs (pooled OR = 0.65, 95% CI 0.46–0.92, *P* = 0.016), substantial heterogeneity was observed within the subgroup (I^2^ = 86.5%). A significant protective effect was also noted for all-cause mortality (7 studies; OR = 0.59, 95% CI 0.39–0.89, *P* = 0.011), with moderate heterogeneity (I^2^ = 53.6%). For CV death (9 studies), no statistical significance was reached (OR = 0.71, 95% CI 0.43–1.18, *P* = 0.187). In the in-hospital acute-phase subgroup, high bilirubin was associated with a significantly increased risk of MACEs (5 studies; OR = 2.33, 95% CI 1.72–3.15, *P* < 0.001), with low heterogeneity (I^2^ = 23.6%). An elevated risk was also noted for CV death (2 studies; OR = 2.79, 95% CI 1.28–6.08, *P* = 0.010). For all-cause mortality (3 studies), the pooled OR was 2.33, but did not reach statistical significance (*P* = 0.084). Between-subgroup heterogeneity tests indicated statistically significant differences in effects (all *P* < 0.01). Further analyses within the long-term follow-up subgroup revealed more pronounced protective effects among older patients (> 65 years), mixed CAD cohorts, and those with a > 70% male proportion. Studies not excluding liver disease showed more robust protective effects, though very few in number. Dose-response analysis showed that no linear or nonlinear association was observed (OR = 0.969, 95% CI 0.919–1.022, *P* = 0.252). Sensitivity analysis based on the leave-one-out method revealed robust pooled results. Begg's test and Egger's test demonstrated that no significant publication bias was detected (all *P* > 0.05).

**Conclusion:**

Serum total bilirubin shows a context-dependent bidirectional association with adverse outcomes in patients with CHD after PCI, but does not demonstrate a consistent independent prognostic value. The apparent protective effect observed in long-term follow-up, older, or stable CHD populations and the risk-elevating effect in the in-hospital acute phase are exploratory findings that require further validation. Given the extremely high heterogeneity (mainly due to inconsistencies in MACE endpoint definitions, liver disease exclusion criteria, and CHD subtype classifications) and the negative dose-response analysis (likely underpowered), current evidence does not support total bilirubin as a robust or generalizable prognostic biomarker. Future prospective studies with standardized designs are needed to elucidate its potential clinical utility.

**Clinical Trial Registration:**

https://www.crd.york.ac.uk/prospero/display_record.php?RecordID=1067176, identifier CRD420251067176.

## 1 Introduction

Coronary heart disease (CHD) refers to a common cardiovascular disease resulting from the stenosis, spasm, or blockage of the coronary arteries, triggering hypoxia, necrosis, or myocardial ischemia. CHD is characterized by sudden onset, severe symptoms, high rates of recurrence and mortality, and multiple complications. The number of patients suffering from CHD increases year by year (1). Percutaneous coronary intervention (PCI), as an important method of revascularization, has significantly relieved the clinical symptoms and improved the short-term prognosis of patients with CHD. Nevertheless, patients with CHD after PCI are still likely to develop long-term cardiovascular events comprising restenosis, myocardial infarction (MI), heart failure, and mortality. Therefore, identifying reliable prognostic biomarkers to optimize risk stratification and individualized treatment strategies has become a significant topic in clinical research.

Bilirubin, a conventional indicator for liver function in recent years, has gradually attracted attention for its potential protective effects on the cardiovascular system. Bilirubin is the terminal metabolite in the catabolism of heme and has been proven to have properties of antioxidation, anti-inflammation, and anti-atherosclerosis. Basic research has shown that bilirubin delays the progression of atherosclerosis by scavenging oxygen-free radicals, suppressing the oxidation of low-density lipoprotein (LDL), and reducing the inflammatory responses of vascular endothelium. Nonetheless, there is a paradox regarding the relation of bilirubin level in the serum with the incidence and progression of CHD (2). Schwertner et al. were the first to investigate this association and found that individuals with a bilirubin level within a high normal range were at a lower risk of CHD. Since then, numerous studies have revealed that bilirubin may be protective; however, some research has reported contradictory findings (3). Huang et al. followed up 544 patients with CHD who underwent PCI for at least 3 years and found that a higher level of bilirubin may be connected to a lower incidence of major adverse cardiovascular events (MACE) (4). However, Yang et al. have revealed that total bilirubin at a higher level at baseline among patients with new-onset non-ST-elevation myocardial infarction (NSTEMI) results in a higher risk of MACE (5). These contradictory results may be triggered by differences in study design, subtypes of CHD, the timing of total bilirubin detection, and the definition of cut-off values. Hence, a systematic analysis is urgently needed to clarify the clinical value of total bilirubin level.

Despite the fact that several observational studies have explored the relation of bilirubin level with the prognosis of PCI, the existing evidence is scattered and inconsistent, lacking systematic reviews of evidence-based medicine with a large sample size. In addition, it is imperative to elucidate the optimal cut-off value for total bilirubin level as a biomarker, its specific ability to predict different clinical endpoints [such as cardiovascular death (CV death), all-cause mortality, and recurrent MI], and its interactions with other conventional risk factors. Against this background, this study, through a meta-analysis, intends to comprehensively investigate the prognostic value of total bilirubin level in the serum for individuals with CHD undergoing PCI, thereby providing a scientific basis for risk stratification and intervention strategies in clinical practice.

## 2 Methods

This study strictly abode by the Preferred Reporting Items for Systematic Reviews and Meta-Analyses (PRISMA) (6) and was registered on the International Prospective Register of Systematic Reviews (PROSPERO) (Registration Number: CRD420251067176) to ensure transparency and reproducibility. The PRISMA 2020 checklist was provided in Supplementary Table 1.

### 2.1 Strategy for literature retrieval

Four major databases comprising PubMed, Cochrane Library, Embase, and Web of Science were searched from the establishment of the databases to May 22, 2026. The search strategy was formulated via the combination of subject terms (MeSH/Emtree) with free terms and through Boolean operators (AND/OR/NOT). The core search terms included ‘bilirubin’ and ‘percutaneous coronary intervention’. The search strategy is detailed in Supplementary Table 2. Additionally, the reference lists of the included studies and pertinent reviews were manually retrieved to supplement any literature that might have been missed.

### 2.2 Inclusion and exclusion criteria

The inclusion criteria were defined in light of the PICOS principle: Participant (P): Patients who were diagnosed with CHD and underwent PCI, regardless of age, sex, or race. Intervention (I): Total bilirubin level in the serum as the exposure variable, with patients categorized into a group with total bilirubin at a high level and a group with total bilirubin at a low level. Comparison (C): Comparison between the group of high-level total bilirubin and the group of low-level total bilirubin. Outcome (O): Studies reporting at least one outcome regarding prognosis, including MACEs, all-cause mortality, and CV death. Study design (S): Case-control studies, cohort studies, or original research published in English.

Studies were excluded provided that they were: (i) review articles, systematic reviews and meta-analyses, conference abstracts, case reports, editorials, or comments without original clinical data; (ii) duplicate publications; (iii) studies from which effect sizes could not be extracted; or (iv) studies involving patients with CHD who did not receive PCI.

### 2.3 Literature screening and data extraction

Literature screening and data extraction were performed by 2 researchers (Zhouxin Wu, Jinchuang Li) independently. The retrieved literature was imported into EndNote X9. The titles and abstracts were reviewed to exclude studies obviously not meeting the inclusion criteria. A full-text review of potentially eligible studies was conducted on the basis of the inclusion and exclusion criteria. The following data were obtained, comprising basic information of the included studies, characteristics of the participants, definition of exposure and control (cut-off value of total bilirubin level), outcome indicators, effect sizes, 95% confidence intervals (CIs), and methodological quality of the selected studies. The collected data were recorded in an Excel spreadsheet and were cross-checked by the two researchers to ensure accuracy. Any disagreement was settled via negotiation with a third researcher (Jing Sun).

### 2.4 Quality evaluation

The methodological quality of the included studies was appraised utilizing the Newcastle-Ottawa Scale (NOS), a tool commonly used for evaluating the quality of case-control studies and cohort studies. The assessment for both types of studies revolved around 3 core domains. Among them, case-control studies were evaluated from 3 aspects: the selection of the case group and the control group, comparability, and exposure assessment. Cohort studies were assessed from 3 dimensions: the selection of cohorts, comparability, and outcome measurement. The maximum score for both was 9 points, and studies scoring ≥7 were generally rated as high quality. Two researchers independently conducted the quality assessment, with any disagreement resolved by a third researcher.

### 2.5 Statistical analysis

STATA 16.0 was leveraged for the statistical analysis, with a significance level of *α*=0.05. The categorical variables were presented using odds ratios (ORs) alongside their 95% CIs, and standardized mean differences (SMDs) along with their 95% CIs were employed to represent continuous variables. In the included studies reporting hazard ratios (HRs) alongside their 95% CIs, ORs were used for the pooled analyses due to the approximation of HR and OR under low event rate in the analyses of the prognosis of PCI. The heterogeneity was assessed using the Cochrane Q test (*P* value) and I^2^ statistic. *P* < 0.1 or I^2^ > 50% signified significant heterogeneity, and a random-effects model was employed; otherwise, a fixed-effects model was adopted. Of note, extremely high between-study heterogeneity was observed in this study. Therefore, the overall pooled results should be interpreted only as trend references rather than as definitive quantitative inferences. Due to the limitations of the available data from the included studies, only ORs could be extracted, and the gold-standard hazard ratio (HR) model for prognosis research was not applicable, constituting an inherent methodological limitation. Given the anticipated clinical heterogeneity between the in-hospital acute-phase setting and the long-term follow-up setting (differences in patients’ pathophysiological status, composition of endpoint indicators, and expected direction of bilirubin effects), all primary analyses were prespecified to be stratified by follow-up duration. The unstratified overall pooled analysis was conducted only for exploratory purposes.

To explore the source of heterogeneity, the stratification analyses were performed based on the following factors:

- According to whether patients with a history of liver disease were excluded, studies were categorized into the group with exclusion of prior liver disease and the group without exclusion of prior liver disease.
- Based on the country in which the study was performed, studies were classified into those conducted in China and those performed in other countries.
- According to the proportion of male participants in the study population, studies were divided into the group with ≥70% male participants and the group with <70% male participants.
- Based on the mean age of the study population, studies were classified into the middle age group (45–65 years) and the older age group (>65 years).
- According to the subtype of CHD, studies were divided into the pure MI group and the mixed CHD group (including patients with stable CHD, unstable angina, MI, and multiple types of CHD).
- Based on the timing of total bilirubin measurement, studies were classified into the preoperative group, the postoperative group, and the mixed group (where measurement was performed either preoperatively or postoperatively without a clear distinction).
- According to the cutoff value used to define high vs. low total bilirubin groups, the median of the cutoff values from the included studies (10.26 μmol/L) was used as the split point, and studies were divided into the relatively low cutoff group (cutoff ≤10.26 μmol/L) and the relatively high cutoff group (cutoff >10.26 μmol/L).
- Based on the follow-up scenario, studies were divided into the in-hospital group (where only in-hospital outcome events were recorded) and the long-term follow-up group (where outcome events during follow-up after discharge were recorded).
- Given the inherent differences in the composition of MACE endpoints between in-hospital and long-term follow-up scenarios — that is, rehospitalization and repeat revascularization cannot be captured during in-hospital follow-up, which only includes acute-phase safety hard endpoints, whereas long-term follow-up can include these effectiveness-related soft endpoints — a prespecified subgroup analysis by follow-up scenario was conducted to eliminate heterogeneity caused by differences in endpoint composition. With reference to (7), studies were classified according to the definition of MACE endpoints into the safety-endpoint MACEs group (including only all-cause or cardiogenic death, MI, stroke, and stent thrombosis) and the composite-endpoint MACEs group (including target vessel or target lesion revascularization, heart failure, rehospitalization, and other events in addition to safety events).

### 2.6 Pre-analysis of heterogeneity in the definition of liver disease

Given that total bilirubin was a direct indicator for liver function, inconsistencies in the definition and exclusion criteria for liver disease across studies were considered a potential major source of between-study heterogeneity. Accordingly, a qualitative comparison of the included studies was planned. The definition of liver disease, the exclusion criteria, and whether biochemical indicators were used for confirmation were recorded for each study (Supplementary Table 3). Nevertheless, due to insufficient reporting and a lack of standardized criteria in the original studies, a quantitative meta-regression analysis could not be performed.

## 3 Results

**Figure 1:**
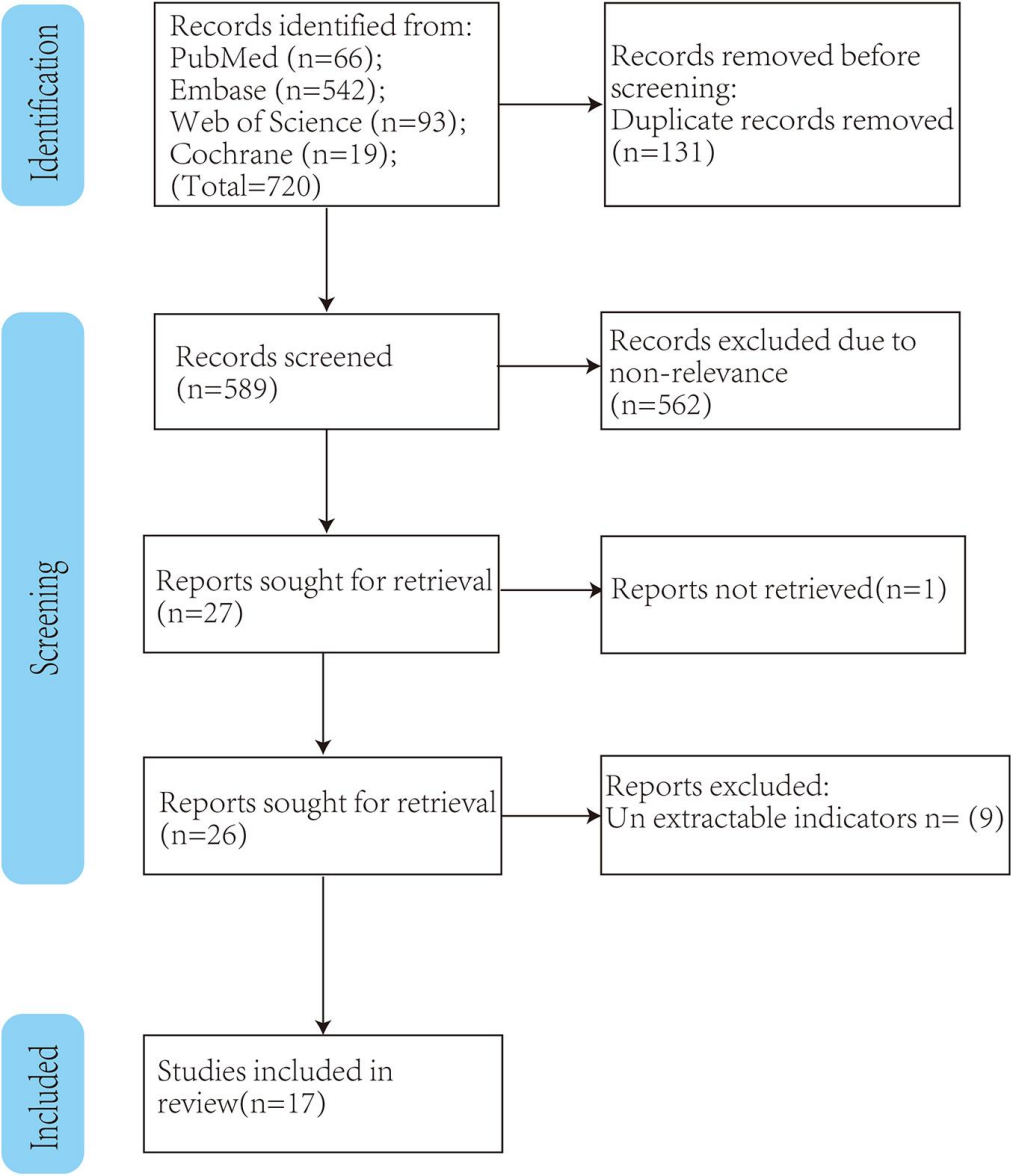
Flow diagram for literature screening.

A meta-analysis was performed adhering to the PRISMA. Originally, 720 records in total were obtained from the 4 databases, with 131 duplicates eliminated. After an initial screening of the remaining 589 studies, 562 irrelevant ones were excluded. A full-text search was carried out on the remaining 27 studies, and 1 study was removed as the full text was unavailable. Data could not be extracted from 9of the remaining 26 studies; they were therefore screened out. Finally, 17 studies in total were included in this study. The details are illustrated in Figure 1.

### 3.1 Characteristics of the included studies

Ultimately, 17 studies in total were included, involving 27,580 patients. The average age ranged from 58 to 70 years, and the proportion of male patients varied widely from 40% to 85%. The majority of the included studies originated from China (*n* = 13), followed by Republic of Korea (*n* = 2), Turkey (*n* = 2). The characteristics are detailed in Table 1.

**Table 1:** Characteristics of the included studies.

First Author	Publication Year	Study Design	Country	Subtype of CHD	Sample Size	Proportion of Male Patients (%)	Age	Follow-Up Duration	Group (*μ*mol/L)	Timing of Measurement	Definition of MACEs	Exclusion of Prior Liver Disease	Outcome
Zhang et al. (22)	2017	Prospective Study and Cohort Study	China	CAD	432	343 (79.4)	61.29 ± 10.17	15 to 51 Months	Control group≤8.53 Exposure group>8.54	**Fasting at admission**	Cardiac death, myocardial infarction, ischemia-driven target vessel revascularization, rehospitalization for unstable angina, rehospitalization for heart failure	Yes	MACEs and All-Cause Mortality
Huang et al. (4)	2012	Retrospective Study and Cohort Study	China	AP or AMI	544	463 (85.1)	70.35 ± 11.79	At Least 3 Years	Control group≤8.55 Exposure group>8.55	**Fasting at admission**	Death, non-fatal myocardial infarction, ischemic stroke	Yes	MACEs, CV Death, and All-Cause Mortality
Fang et al. (23)	2022	Retrospective Study and Case-control Study	China	STEMI	466	366 (78.5)	60.99 ± 4.85	NA	NA	**At admission**	Cardiac death, recurrent myocardial infarction (stent thrombosis), malignant arrhythmia, and acute heart failure	Yes	MACEs
Li et al. (13)	2022	Retrospective Study and Cohort Study	China	MI	10,236	7,311 (71.4)	66.27 ± 10.23	Median Duration of Follow-Up: 3.2 Years	Control group≤10.2 Exposure group>10.2	**Pre-PCI**	Cardiac death, myocardial infarction, stroke, revascularization	Yes	MACEs and CV Death
Gao et al. (24)	2019	Prospective Study and Cohort Study	China	ACS and SA	2,502	1,701 (68.0)	59.9 ± 11.1	3 Years	Control group≤10.26 Exposure group>10.26	**At admission**	CV death, non-fatal myocardial infarction, stroke	Yes	MACEs and CV Death
Chung et al. (25)	2016	Retrospective Study and Cohort Study	South Korea	STEMI	1,111	832 (74.9)	62.5 ± 12.4	12 Months	Control group≤13.51 Exposure group>13.51	**Peri-PCI**	Cardiac death, non-fatal myocardial infarction, definite or probable stent thrombosis	Yes	MACEs and CV Death
Acet et al. (26)	2014	Retrospective Study and Cohort Study	Turkey	STEMI	360	262 (72.8)	61.39 ± 13.7	NA	Control group≤14.11 Exposure group>14.11	**Pre-PCI**	Acute stent thrombosis, cardiogenic shock, new or progressive heart failure, pulmonary edema, complete atrioventricular block, severe ventricular arrhythmia, major bleeding	Yes	MACEs and All-Cause Mortality
Tuxun et al. (27)	2020	Prospective Study and Case-control Study	China	STEMI	615	525 (85.4)	58.22 ± 12.19	30.2 Months on Average	NA	**At admission**	Cardiac death, rehospitalization for angina, non-fatal myocardial infarction, malignant arrhythmia, severe cardiac dysfunction, in-stent restenosis, target vessel revascularization	Yes	MACEs
Ying et al. (28)	2023	Prospective Study and Cohort Study	China	STEMI	418	167 (40)	59.23 ± 12.98	NA	Control group<10.69 Exposure group≥10.69	**Post-PCI**	Post-infarction angina, recurrent myocardial infarction, acute heart failure, cardiogenic shock, malignant arrhythmia, death	Yes	MACEs
Yao et al. (29)	2015	Prospective Study and Cohort Study	China	AP	1,419	931 (65.6)	60.9 ± 10.5	12 Months	Control group≤8.38 Exposure group>8.38	**At admission**	Death, myocardial infarction, stroke	Yes	MACEs, CV Death, and All-Cause Mortality
Gul et al. (30)	2013	Retrospective Study and Cohort Study	Turkey	STEMI	1,624	1,341 (82.6)	56.82 ± 11.65	Average Duration of Follow-Up: 26.2 Months	Control group≤15.39 Exposure group>15.39	**Post-PCI**	CV death, reinfarction, repeat target vessel revascularization	Yes	MACEs and CV Death
Zhao et al. (31)	2020	Retrospective Study and Cohort Study	China	STEMI	3,708	2,909 (78.5)	59.13 ± 0.58	Median Duration of Follow-Up: 754 Days	Control group≤22 Exposure group≥22	**At admission or Post-PCI**	All-cause death, cardiac death, recurrent myocardial infarction, ischemic stroke	No	MACEs, CV Death, and All-Cause Mortality
Wei et al. (32)	2014	Retrospective Study and Case-control Study	China	AMI	239	193 (81)	59.9 ± 11.2	3 Years	NA	**At admission**	All-cause death, non-Q-wave myocardial infarction, Q-wave myocardial infarction, target vessel revascularization	No	MACEs
Zhang et al. (33)	2014	Prospective Study and Cohort Study	China	ACS	1,152	782 (67.9)	60.8 ± 10.8	30 ± 5 Months	Control group≤10.7 Exposure group＞10.7	**Pre-PCI**	All-cause death, myocardial infarction, repeat revascularization, and rehospitalization	Yes	MACEs and All-Cause Mortality
Yang et al. (5)	2022	Prospective Study and Cohort Study	China	NSTEMI	327	217 (66.4)	60.36 ± 10.98	30.33 Months on Average	Control group<10.23 Exposure group≥10.23	**Pre-PCI**	Cardiac death, myocardial infarction, stent thrombosis, stroke, revascularization	Yes	MACEs and All-Cause Mortality
Kim et al. (34)	2015	Retrospective Study and Cohort Study	South Korea	CAD	372	235 (63.2)	65.2 ± 10.7	774 ± 359 Days	Control group<9.92 Exposure group≥9.92	**Pre-PCI**	Cardiac death, myocardial infarction, target vessel revascularization, ischemic stroke, stent thrombosis	No	MACEs and CV Death
Yu et al. (35)	2026	Retrospective Study and Cohort Study	China		2,055	1,654 (80)	60.9 ± 9.8	Median Duration of Follow-Up: 36 Months	Control group<12.39 Exposure group≥12.39	**At admission**	all-cause mortality, myocardial infarction, stroke, repeat revascularization, and stent thrombosis.	Yes	MACEs, CV Death, and All-Cause Mortality

### 3.2 Quality evaluation

Overall, a score of ≥7 stars was considered to indicate high quality. The thirteen cohort studies were assessed for quality, with scores ranging from 7 to 9. The three case-control studies each received a score of 7. The overall quality of the included studies was relatively high. The details are shown in Tables 2, 3.

**Table 2:** Criteria of quality evaluation for the cohort studies using the Newcastle-Ottawa scale.

Study	Selection				Comparability	Outcome			Quality scores
	Representativeness of the exposed cohort	Selection of the nonexposed cohort	Ascertainment of exposure	Demonstration that the outcome of interest was not present at the start of the study	Comparability of cohorts based on the design or analysis	Assessment of outcome	Was the follow-up long enough for outcomes to occur	Adequacy of follow-up of cohorts	
Zhang et al. (22)	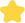	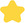	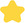	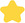	**-**	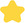	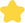	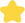	7
Huang et al. (4)	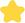	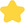	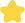	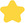	**-**	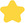	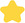	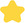	7
Li et al. (13)	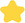	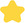	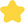	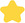	**-**	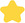	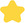	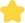	7
Gao et al. (24)	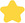	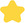	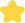	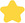	**-**	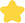	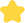	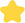	7
Chung et al. (25)	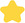	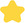	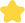	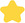	**-**	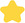	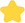	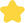	7
Acet et al. (26)	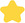	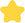	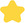	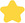	**-**	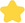	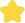	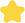	7
Ying et al. (28)	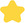	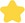	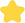	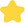	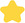 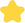	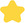	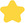	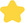	9
Yao et al. (29)	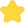	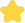	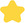	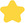	**-**	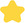	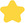	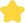	7
Gul et al. (30)	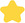	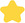	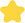	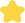	**-**	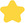	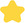	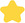	7
Zhao et al. (31)	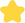	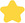	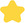	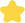	**-**	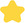	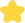	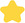	7
Zhang et al. (33)	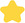	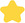	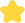	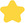	**-**	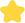	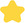	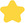	7
Yang et al. (5)	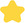	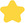	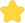	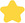	**-**	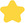	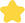	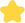	7
Kim et al. (34)	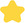	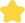	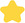	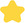	**-**	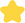	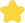	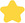	7
Yu et al. (35)	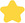	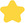	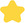	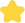	**-**	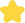	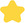	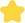	7

**Table 3:** Criteria of quality evaluation for the case-control studies using the Newcastle-Ottawa scale.

study	Selection				Comparability	Exposure			Overall score
	Is the case definition adequate?	Representativeness of the cases	Selection of controls	Definition of Controls	Comparability of cohorts based on the design or analysis	Ascertainment of exposure	Same method of ascertainment for cases and controls	non-response rate	
Fang et al. (23)	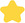	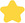	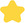	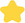	-	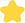	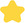	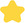	7
Tuxun et al. (27)	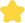	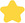	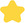	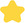	-	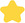	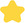	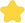	7
Wei et al. (32)	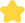	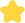	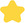	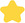	-	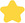	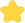	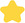	7

### 3.3 Meta-analysis

#### 3.3.1 Primary stratification analysis by follow-up setting

**Figure 2:**
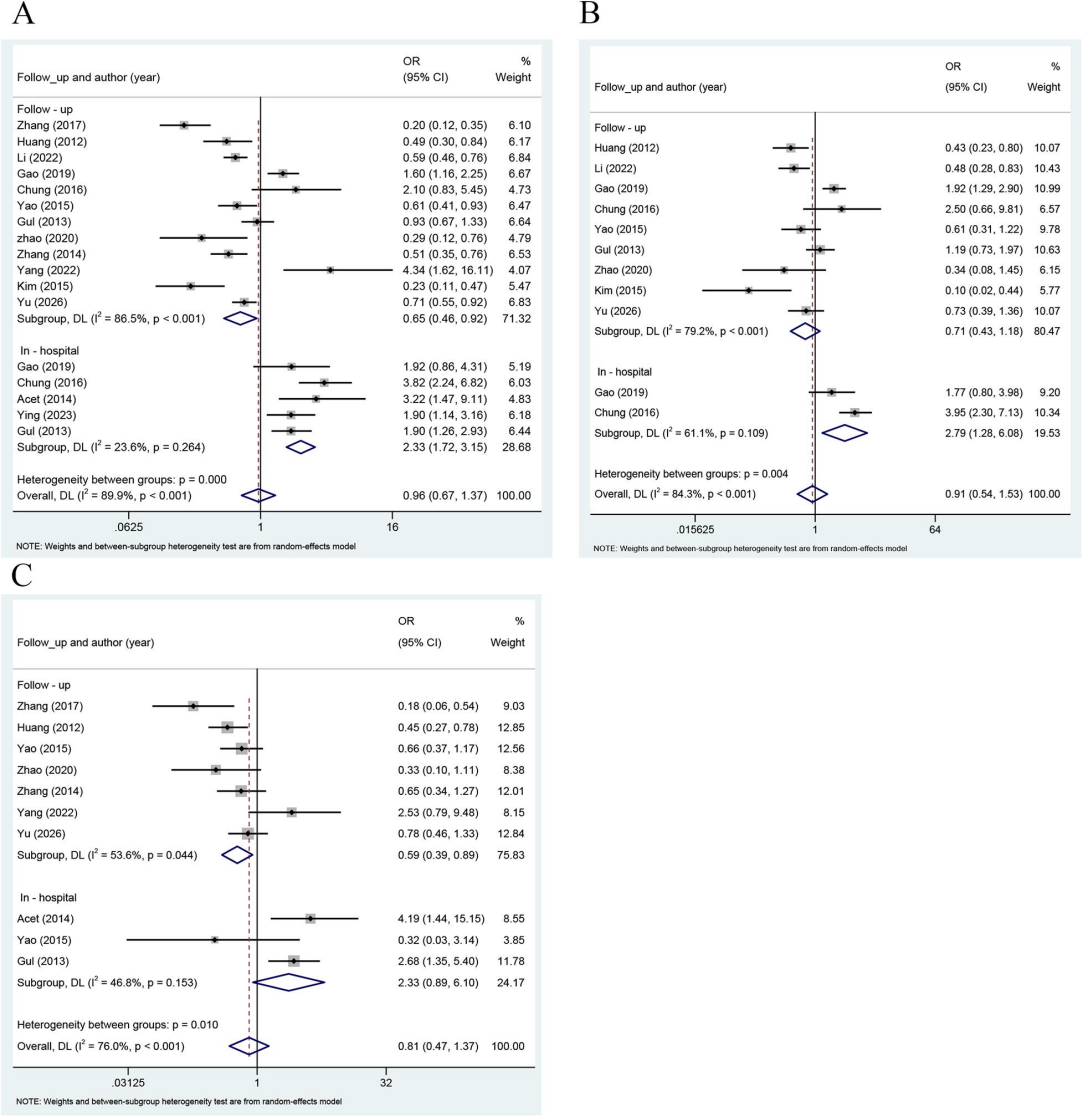
**(A)** Subgroup analysis of the association between serum total bilirubin and risk of MACEs: long-term follow-up vs. in-hospital analysis (binary OR); **(B)** subgroup analysis of the association between serum total bilirubin and risk of CV death: long-term follow-up vs. in-hospital analysis; **(C)** subgroup analysis of the association between serum total bilirubin and risk of all-cause mortality: long-term follow-up vs. in-hospital analysis.

The pathophysiological roles of bilirubin differ completely between acute inflammatory stress and chronic stable conditions. Accordingly, clinical status was designated as the primary *a priori* stratification factor in this study. All primary analyses were performed independently after stratification to avoid bias resulting from the confounding of different pathophysiological statuses. In accordance with the prespecified analysis plan, follow-up setting (long-term follow-up vs. in-hospital acute-phase) was defined as a key potential source of heterogeneity. Given the fundamental differences in endpoint composition and patients’ pathophysiological status between the two settings, mutually exclusive stratification analyses were performed *a priori*. The key statistical results are summarized in Table 4 and Figures 2A–C.

**Table 4:** Summary of core statistical results from subgroup analysis stratified by follow-up scenario for the association between Serum total bilirubin and adverse outcomes Among patients with CHD after PCI.

Outcome Indicator	Subgroup Type	Number of Included Studies	Pooled OR (95% CI)	*P* Value for Effect Size	I^2^ for Within-Subgroup Heterogeneity Test	*P* Value for Within-Subgroup Heterogeneity Test	*P* Value for Between-Subgroup Heterogeneity Test
MACEs	Long-term Follow-up Subgroup	12	0.65 (0.46–0.92) ^*^	0.016	86.5%	<0.001	<0.001
	In-hospital Acute Phase Subgroup	5	2.33 (1.72–3.15)^*^	<0.001	23.6%	0.264	<0.001
CV Death	Long-term Follow-up Subgroup	9	0.71 (0.43–1.18)	0.187	79.2%	<0.001	0.004
	In-hospital Acute Phase Subgroup	2	2.79 (1.28–6.08)^*^	0.010	61.1%	0.109	0.004
All-cause Mortality	Long-term Follow-up Subgroup	7	0.59 (0.39–0.89)^*^	0.011	53.6%	0.044	<0.001
	In-hospital Acute Phase Subgroup	3	2.33 (0.89–6.10)	0.084	46.8%	0.153	<0.001

* Denotes statistical significance of the pooled effect (*P* < 0.05).

The primary stratified meta-analysis results according to the follow-up setting are presented in Table 4. For MACEs, 12 studies reported long-term follow-up data, and 5 studies reported data from the in-hospital acute phase. In the long-term follow-up subgroup, a higher serum total bilirubin level was significantly associated with a lowered risk of MACEs (pooled OR = 0.65, 95% CI: 0.46–0.92, *P* = 0.016), although within-subgroup heterogeneity remained high (I^2^ = 86.5%, *P* < 0.001). In contrast, among patients followed during the in-hospital acute phase, a higher bilirubin level was associated with a significantly increased risk of MACEs (OR = 2.33, 95% CI: 1.72–3.15, *P* < 0.001), with low heterogeneity (I^2^ = 23.6%, *P* = 0.264). The between-subgroup difference was highly significant (*P* < 0.001), confirming that the direction of association is fully opposite in the two clinical settings.

For CV death, 9 studies contributed to the long-term follow-up analysis and 2 studies to the in-hospital acute-phase analysis. No significant association was observed in the long-term follow-up subgroup (OR = 0.71, 95% CI: 0.43–1.13, *P* = 0.201), with substantial heterogeneity (I^2^ = 79.2%, *P* < 0.001). In contrast, in the in-hospital acute-phase subgroup, a higher bilirubin level was significantly associated with an elevated risk of CV death (OR = 2.79, 95% CI: 1.28–6.08, *P* = 0.010), with moderate heterogeneity (I^2^ = 61.1%, *P* = 0.109). The between-subgroup heterogeneity was significant (*P* = 0.005).

For all-cause mortality, 7 studies were available for long-term follow-up and 3 studies for in-hospital acute-phase. A higher bilirubin level was associated with a significantly lower risk of all-cause mortality in the long-term follow-up subgroup (OR = 0.60, 95% CI: 0.39–0.90, *P* = 0.018), with moderate heterogeneity (I^2^ = 57.6%, *P* = 0.044). In the in-hospital acute-phase subgroup, the pooled OR was 2.33, but the association did not reach statistical significance (95% CI: 0.89–6.10, *P* = 0.085), and heterogeneity was low to moderate (I^2^ = 46.8%, *P* = 0.153). The between-subgroup difference was significant (*P* = 0.009).

Table 4 comprehensively presents the biphasic effect patterns, effect sizes, statistical significance, and within- and between-group heterogeneity tests for the association between a higher serum total bilirubin level and the three core endpoints across different follow-up settings. It was clearly confirmed that the divergent effect directions between the two settings did not represent contradictory results; instead, they reflected differences in true effects under distinct clinical scenarios, with solid statistical support.

#### 3.3.2 Overall unstratified analysis

To avoid potential bias caused by multiple follow-up time points from the same study, only the data with the longest follow-up duration were extracted from each study for the overall pooled analysis (without distinguishing between long-term follow-up and in-hospital acute-phase settings). Pooled analyses were performed for the three outcomes. The results showed a pooled OR of 0.97 for MACEs (95% CI 0.68–1.37, *P* > 0.05), with extremely high heterogeneity (I^2^ = 88.7%, *P* < 0.001); a pooled OR of 0.71 for CV death (95% CI 0.43–1.18, *P* > 0.05), with substantial heterogeneity (I^2^ = 79.2%, *P* < 0.001); and a pooled OR of 0.84 for all-cause mortality (95% CI 0.48–1.45, *P* > 0.05), with substantial heterogeneity (I^2^ = 78.4%, *P* < 0.001). In addition, after a continuous variable analysis for MACEs (comparing bilirubin levels between patients with and without MACEs), a pooled SMD of −0.37 (95% CI −0.77 to 0.02, *P* > 0.05) was observed, with extremely high heterogeneity (I^2^ = 94.0%, *P* < 0.001).

The overall unstratified pooled results were not statistically significant and exhibited extremely high heterogeneity, indicating no stable association between the variables.，Moreover, the overall pooled heterogeneity was extremely high for all outcomes (I^2^ > 78%), further indicating that simple pooling does not conform to statistical principles. The overall pooled forest plots are presented in Supplementary Figures 1A–C, and the forest plot for the continuous variable difference is provided in Supplementary Figure 1D.

#### 3.3.3 Additional analyses within the long-term follow-up subgroup

Stratification analyses were performed according to prespecified factors to further explore the sources of high heterogeneity (I^2^ = 86.5%) within the long-term follow-up subgroup. At the level of country or region, although a borderline significant association between high bilirubin and a reduced risk of MACEs was observed in the 10 studies performed in China (OR = 0.69, 95% CI 0.47–1.01, *P* = 0.059), within-subgroup heterogeneity remained high (I^2^ = 87.1%). The only study from the Republic of Korea showed the strongest protective effect (OR = 0.23, 95% CI 0.12–0.44), while no significant association was found in the Turkish studies. When stratified by CHD subtype, the analysis indicated that a significantly lowered risk of MACEs associated with high bilirubin was observed in the mixed CAD cohort (6 studies; OR = 0.50, 95% CI 0.28–0.89, *P* = 0.019), with extremely high heterogeneity (I^2^ = 91.4%). No significant association was found in the isolated AMI group (6 studies; OR = 0.85, 95% CI 0.54–1.33, *P* = 0.476), with heterogeneity of 78.5%. The stratification analysis by age revealed that a significantly reduced risk of MACEs associated with high bilirubin was noted only in the older age subgroup (>65 years, 3 studies; OR = 0.45, 95% CI 0.28–0.72, *P* = 0.001), and heterogeneity decreased markedly to 65.9% (*P* = 0.053). No statistical significance was reached in the middle-aged group (45–65 years, 9 studies; OR = 0.77, 95% CI 0.49–1.19, *P* = 0.239), with heterogeneity of 88.2%. The stratification analysis by proportion of male patients revealed that a significantly lowered risk of MACEs associated with high bilirubin was demonstrated in the 7 studies with >70% male participants (OR = 0.58, 95% CI 0.39–0.85, *P* = 0.021), with heterogeneity of 82.1%. No significant association was observed in the 5 studies with ≤70% male participants (OR = 0.81, 95% CI 0.39–1.67, *P* = 0.564), with heterogeneity as high as 90.8%. Regarding the definition of MACE endpoints, a significantly reduced risk of MACEs associated with high bilirubin was observed in the 8 studies that adopted a composite endpoint (including soft endpoints) (OR = 0.54, 95% CI 0.37–0.80, *P* = 0.002), with heterogeneity of 84.5%. No significant association was found in the 4 studies that used only safety hard endpoints (OR = 0.96, 95% CI 0.49–1.88, *P* = 0.898), with heterogeneity of 87.0%. When stratified by total bilirubin cutoff value, the analysis revealed that neither the relatively low bilirubin group (cutoff ≤10.26 μmol/L, 7 studies) nor the relatively high bilirubin group (cutoff >10.26 μmol/L, 5 studies) reached statistical significance (*P* = 0.105 and *P* = 0.085, respectively), and both groups exhibited extremely high heterogeneity (I^2^ = 93.6% and 86.5%, respectively). Regarding excluding the history of liver disease, a protective effect of high bilirubin that approached borderline significance was noted in the 10 studies that excluded liver disease (OR = 0.73, 95% CI 0.53–1.07, *P* = 0.117), with heterogeneity of 87.0%. A stronger protective effect was shown in the 2 studies not excluding liver disease (OR = 0.25, 95% CI 0.14–0.44, *P* < 0.001), with no heterogeneity (I^2^ = 0%). Nevertheless, this finding should be interpreted with caution due to the extremely small number of included studies and the potential for residual confounding (e.g., uncorrected abnormal liver function). When stratified by the timing of bilirubin measurement, the analysis demonstrated that a significantly lowered risk of MACEs associated with high bilirubin was shown in the preoperative measurement subgroup (9 studies; OR = 0.61, 95% CI 0.41–0.91, *P* = 0.016), with heterogeneity of 88.4%. No statistical significance was reached in the postoperative measurement group (2 studies) or the mixed timing group (1 study). In summary, age, the composition of MACE endpoints, CHD subtype, proportion of male patients, and exclusion of prior liver disease were identified as important sources of heterogeneity within the long-term follow-up subgroup. Nonetheless, even after the above stratifications, heterogeneity remained high in most subgroups (I^2^ > 80%), suggesting that other unmeasured factors (e.g., study-specific definitions of liver disease, inflammatory levels, and renal function) may continue to exert confounding effects. The characteristics are detailed in Table 5.

**Table 5:** Prespecified subgroup analysis of the association between total bilirubin and MACEs during long-term follow-up.

Total Bilirubin Level and MACEs (Follow-up)	Number of Included Studies	OR and 95% CI	P	I^2^and P
Country				
China	10	0.69 (0.47, 1.01)	0.059	I^2^ = 87.10%, *P* < 0.001
Turkey	1	1.85 (0.67,5.10)	0.678	I^2^= 0.0%. *P* < 0.001
South Korea	1	0.23 (0.12,0.44)	<0.001	I^2^ = 0.0%, *P* < 0.001
Subtype of CHD				
Mixed CAD Cohort Group	6	0.50 (0.28, 0.89)^*^	0.019	I^2^ = 91.4%, *P* < 0.001
Isolated AMI Group	6	0.85 (0.54–1.33)	0.476	I^2^ = 78.5%, *P* < 0.001
Age				
Middle Age Group (45–65 years old)	9	0.77 (0.49, 1.19)	0.239	I^2^ = 88.2%, *P* < 0.001
Older Age Group (>65 years old)	3	0.45 (0.28, 0.72)^*^	0.001	I^2^ = 65.9%, *P* = 0.053
Proportion of Male Patients				
>70%	7	0.58 (0.39, 0.85)^*^	0.021	I^2^ = 82.1%, *P* < 0.001
≤70%	5	0.81 (0.39, 1.67)	0.564	I^2^ = 90.8%, *P* < 0.001
MACEs grouping				
Composite endpoint MACEs group	8	0.54 (0.37,0.80)^*^	0.002	I^2^ = 84.5%, *P* < 0.001
Safety-only MACEs group	4	0.96 (0.49,1.88)	0.898	I^2^ = 87%, *P* < 0.001
Total bilirubin cutoff				
Low cutoff group	7	0.62 (0.35, 1.11)	0.105	I^2^ = 93.6%, *P* < 0.001
High cutoff group	5	0.72 (0.49, 1.05)	0.085	I^2^ = 86.5%, *P* < 0.001
Exclusion of prior liver disease				
Liver disease excluded	10	0.73 (0.53.1.07)	0.117	I^2^ = 87.0%, *P* < 0.001
Liver disease not excluded	2	0.25 (0.14, 0.44)	<0.001	I^2^ = 0.0%, *P* = 0.001
Timing of Measurement				
Preoperative bilirubin subgroup	9	0.61 (0.41, 0.91)^*^	0.016	I^2^ = 88.4%, *P* < 0.001
Mixed timing subgroup	1	2.10 (0.82,5.38)	0.122	I^2^ = 0%, *P* < 0.001
Postoperative bilirubin	2	0.56 (0.18, 1.75)	0.320	I^2^ = 81.4%, *P* < 0.001

^*^Denotes statistical significance of the pooled effect (*P* < 0.05).

#### 3.3.4 Dose-response meta-analysis for long-term follow-up MACEs

Six studies were included in the dose-response analysis. A restricted cubic spline model with four nodes (1.7, 8.35, 13.68, and 20.5 μmol/L) was used to assess the nonlinear relationship. The test for nonlinearity was not significant (*χ*^2^ = 0.31, *P* = 0.855), indicating that a linear model could describe the dose-response relationship. In the linear dose-response model, the pooled OR for MACEs risk per 1 μmol/L increase in serum total bilirubin was 0.969 (95% CI 0.919–1.022, *P* = 0.252), corresponding to an approximately 3% reduction in risk, which was not statistically significant. The between-study variance (*τ*^2^) was 0.0038, indicating moderate heterogeneity. Due to the limited number of included studies (only six) and the wide CIs, the power of this dose-response analysis was insufficient to detect a potential linear trend. Therefore, no credible dose-dependent association was observed between serum total bilirubin levels and the long-term risk of MACEs after PCI. Details are shown in Supplementary Figure 2.

#### 3.3.5 Further subgroup analyses for CV death and All-cause mortality (within the long-term follow-up subgroup)

To further explore the sources of heterogeneity for CV death and all-cause mortality within the long-term follow-up subgroup, additional stratified analyses were performed separately for these two outcomes based on CHD subtype, age, proportion of male patients, timing of bilirubin measurement, cutoff value, and history of liver disease exclusion, as illustrated in Tables 6, 7.

**Table 6:** Prespecified subgroup analysis of the association between total bilirubin and CV death during long-term follow-up.

Total Bilirubin Level and CV Death	Number of the Included Studies	OR and 95% CI	P	I^2^ and P
CHD Subtype				
Mixed CAD Cohort Group	4	0.59 (0.22, 1.59)	0.297	I^2^ = 88.7%, *P* < 0.001
Isolated AMI Group	5	0.76 (0.44, 1.33)	0.342	I^2^ = 61.7%, *P* = 0.033
Age				
Middle Age Group (45–65 years old)	6	1.04 (0.63, 1.70)	0.891	I^2^= 67.5%, *P* = 0.009
Older Age Group (>65 years old)	3	0.38 (0.21, 0.68)*	0.001	I^2^ = 43.6%, *P* = 0.170
Proportion of Male Patients				
>70%	6	0.70 (0.44, 1.13)	0.147	I^2^ = 61.5%,*P* = 0.024
≤70%	3	0.59 (0.16, 2.23)	0.440	I^2^ = 89.4%,*P* < 0.001
Total bilirubin cutoff				
Low cutoff group	5	0.55 (0.24, 1.25)	0.154	I^2^ = 87.7%, *P* < 0.001
High cutoff group	4	0.96 (0.55, 1.67)	0.879	I^2^ = 43.8%, *P* = 0.148
Exclusion of prior liver disease				
Liver disease excluded	7	0.87 (0.53, 1.43)	0.577	I^2^ = 79.3%, *P* < 0.001
Liver disease not excluded	2	0.19 (0.06, 0.63)*	0.007	I^2^ = 22.0%, *P* = 0.258
Timing of Measurement				
Preoperative bilirubin subgroup	6	0.59 (0.31, 1.15)	0.122	I^2^ = 84.7%, *P* < 0.001
Mixed timing subgroup	1	2.50 (0.65, 9.64)	0.183	I^2^ = 0%, *P* < 0.001
Postoperative bilirubin	2	0.77 (0.24,2.48)	0.663	I^2^ = 61.1%, *P* = 0.109

CV Death, cardiovascular death; CHD, coronary heart disease; CAD, coronary artery disease; MI, Myocardial Infarction.

**Table 7:** Prespecified subgroup analysis of the association between total bilirubin and All-cause mortality during long-term follow-up.

Total Bilirubin Level and All-Cause Mortality	Number of the Included Studies	OR and 95% CI	P	I^2^ and P
CHD Subtype				
Mixed CAD Cohort Group	4	0.52 (0.32,0.83)^*^	0.007	I^2^ = 53.4%, *P* = 0.092
Isolated AMI Group	3	0.79 (0.30, 2.05)	0.622	I^2^ = 64.5%, *P* = 0.060
Proportion of Male Patients				
>70%	4	0.44 (0.25, 0.78)^*^	0.005	I^2^ = 54.7%, *P* = 0.085
≤70%	3	0.84 (0.45, 1.58)	0.590	I^2^ = 50.6%, *P* = 0.132
Timing of Measurement				
Preoperative bilirubin subgroup	5	0.50 (0.33, 0.77)^*^	0.001	I^2^= 44.8%, *P* = 0.123
Postoperative bilirubin	2	1.15 (0.31,4.30)	0.832	I^2^ = 72.1%, *P* = 0.058
Total bilirubin cutoff				
Low cutoff group	4	0.57 (0.27,1.20)	0.141	I^2^= 72.0%, *P* = 0.013
High cutoff group	3	0.67 (0.45, 0.99)^*^	0.043	I^2^ = 0.0%, *P* = 0.438
Exclusion of prior liver disease				
Liver disease excluded	6	0.63 (0.40,0.96)^*^	0.032	I^2^ = 58.2%, *P* = 0.035
Liver disease not excluded	1	0.33 (0.10,1.10)	0.071	I^2^ = 0%, *P* < 0.001

All-Cause Mortality, total mortality; CHD, coronary heart disease; CAD, coronary artery disease; MI, myocardial infarction.

^*^Denotes statistical significance of the pooled effect (*P* < 0.05).

For CV death (9 studies), the subgroup analyses showed that although no significant association between high bilirubin and CV death was observed in either the mixed CAD cohort (4 studies) or the isolated AMI group (5 studies) (OR = 0.59 and 0.76, respectively, both *P* > 0.05), heterogeneity was extremely high in the mixed CAD cohort (I^2^ = 88.7%). The stratification analysis by age revealed that a significant reduction in CV death risk associated with high bilirubin was observed only in the older age subgroup (>65 years, 3 studies) (OR = 0.38, 95% CI 0.21–0.68, *P* = 0.001), with moderate to low heterogeneity (I^2^ = 43.6%). When stratified by the proportion of male patients, the analysis revealed that neither the >70% group (6 studies) nor the ≤70% group (3 studies) reached statistical significance. The stratification analysis by bilirubin cutoff value indicated that heterogeneity was markedly reduced in the high-cutoff group (>10.26 μmol/L, 4 studies) (I^2^ = 43.8%); however, no significant association was noted. Regarding exclusion of prior liver disease, no significant association was found in the 7 studies excluding liver disease (OR = 0.87, *P* = 0.577), whereas the 2 studies that did not exclude liver disease reported a stronger protective effect (OR = 0.19, 95% CI 0.06–0.63, *P* = 0.007). However, this finding should be interpreted with caution due to the extremely small number of studies.

For all-cause mortality (7 studies), subgroup analyses revealed that in the mixed CAD cohort (4 studies), high bilirubin was significantly associated with a lowered risk of all-cause mortality (OR = 0.52, 95% CI 0.32–0.83, *P* = 0.007), with moderate heterogeneity (I^2^ = 53.4%). No significant association was observed in the isolated AMI group (3 studies). A significant protective effect (OR = 0.44, 95% CI 0.25–0.78, *P* = 0.005) was noted for the 4 studies with >70% male participants, whereas no significant association was found in the ≤70% group. The 5 studies with preoperative bilirubin measurement showed significant protection (OR = 0.50, 95% CI 0.33–0.77, *P* = 0.001), while no significant association was observed in the postoperative measurement group. The high-cutoff group (>10.26 μmol/L, 3 studies) showed significant protection (OR = 0.67, 95% CI 0.45–0.99, *P* = 0.043) with no heterogeneity (I^2^ = 0%). The low-cutoff group did not reach statistical significance. The 6 studies that excluded liver disease showed significant protection (OR = 0.63, 95% CI 0.40–0.96, *P* = 0.032), while no significant association was noted for the single study not excluding liver disease (OR = 0.33, 95% CI 0.10–1.10, *P* = 0.071), with extremely low strength of evidence.

In summary, age (>65 years) was identified as a significant source of heterogeneity for CV death within the long-term follow-up subgroup. Mixed CAD cohort, male proportion >70%, preoperative bilirubin measurement, and a high bilirubin cutoff value were identified as important effect modifiers for the protective association with all-cause mortality. Studies that excluded liver disease exhibited a more consistent protective effect for both outcomes. Nevertheless, given the extremely limited number of studies without exclusion of prior liver disease, these conclusions should be regarded as exploratory.

#### 3.3.6 Sensitivity analysis and publication bias

To assess the robustness of the pooled results within the long-term follow-up subgroup, a l sensitivity analysis based on the leave-one-out method was performed for MACEs, CV death, and all-cause mortality. The results showed that after sequentially omitting any single study, the direction and significance of the pooled effect sizes for each outcome did not alter substantially (Supplementary Figures 3A-C), indicating the favorable robustness of the above results.

**Figure 3:**
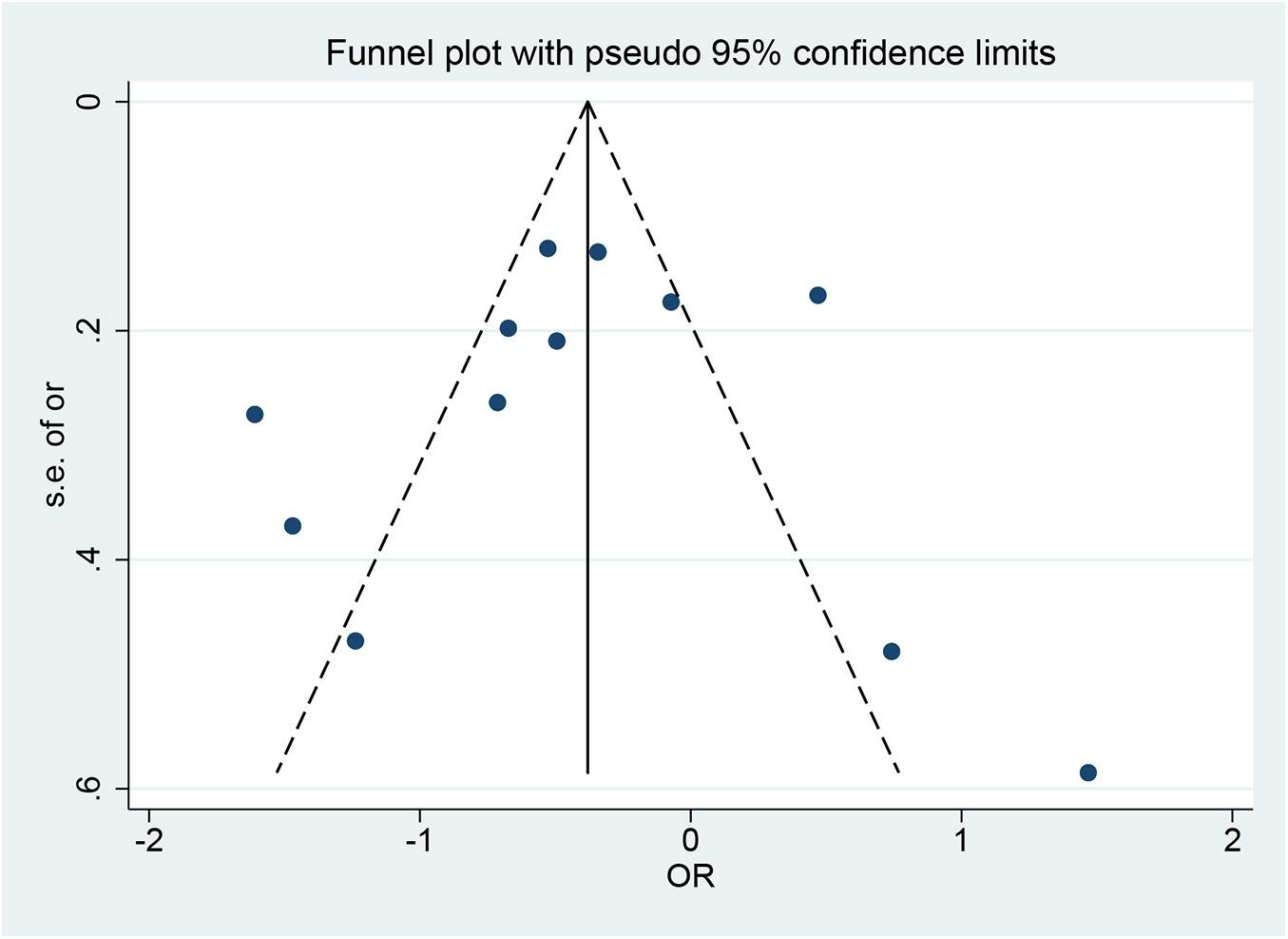
Funnel plot for publication bias of MACEs in the long-term follow-up subgroup.

Publication bias was assessed using Begg's test and Egger's test. For MACEs, neither Begg's test (adjusted Pr > |z| = 0.732) nor Egger's test (*P* for bias = 0.783) indicated significant publication bias (Figure 3). For CV death, Begg's test (adjusted Pr > |z| = 0.175) and Egger's test (*P* for bias = 0.157) also did not reach statistical significance. For all-cause mortality, neither Begg's test (adjusted Pr > |z| = 1.000) nor Egger's test (*P* for bias = 0.857) suggested publication bias. Due to the limited number of included studies (9 for CV death and 7 for all-cause mortality), the power of the publication bias tests was constrained. Nonetheless, no significant bias was detected. Given the extremely small number of studies in the in-hospital acute-phase subgroup, sensitivity analysis and publication bias testing were not performed for that subgroup.

#### 3.3.7 Meta-regression analysis

To additionally explore potential sources of between-study heterogeneity, univariable meta-regression analyses were performed separately for MACEs, CV death, and all-cause mortality within the long-term follow-up subgroup. The covariates included mean age, CHD subtype (mixed CAD vs. isolated AMI), MACE endpoint definition (composite endpoint vs. safety endpoint), total bilirubin cutoff value (as a continuous variable), and exclusion of prior liver disease (yes vs. no). The results are detailed in Supplementary Tables 4–S6. Univariable meta-regression analyses failed to identify any covariate that significantly explained the heterogeneity within the long-term follow-up subgroup. Although mean age and exclusion of prior liver disease exhibited some explanatory power for CV death (adjusted R^2^ approximately 32%–42%), neither reached statistical significance, and the other covariates performed poorly. This suggested that confounding factors unmeasured currently (e.g., study-specific definitions of liver disease, inflammatory levels, and renal function) remained the primary sources of residual heterogeneity.

#### 3.3.8 Additional analysis using hazard ratios (HR)

Although most included studies reported only odds ratios (OR), three studies reported hazard ratios (HR) derived from Cox regression, which better account for time-to-event data. Among these, one study (25) focused on the in-hospital acute-phase population and reported an HR of 2.69 (95% CI 1.67–4.34) for MACEs, indicating an increased risk with higher bilirubin. The other two studies (13, 31) were from the long-term follow-up setting and showed HRs of 0.667 (0.485–0.918) and 0.28 (0.09–0.88), respectively, both suggesting a protective effect. Despite the small number of HR-based studies precluding a formal meta-analysis, the direction of these HR estimates is consistent with our primary stratified OR results, supporting the robustness of the bidirectional prognostic pattern.

## 4 Discussion

The core contribution of this study is that, through a large-scale, *a priori* stratified meta-analysis, the long-standing ‘bilirubin-CHD paradox’ has been systematically explained for the first time among patients after PCI. The contradictory conclusions drawn by previous studies are fundamentally attributed to the failure to distinguish between different clinicopathological stages of CHD (acute stress state vs. chronic stable state) and different follow-up settings (in-hospital acute-phase vs. long-term follow-up). This issue was addressed in this study through the following innovative design: *a priori* stratification by follow-up setting was performed for the first time, and studies were strictly classified into two mutually exclusive subgroups (‘in-hospital acute-phase’ and ‘long-term follow-up’), thereby avoiding the cancellation of effects with opposite directions. On this basis, the bidirectional prognostic effect of bilirubin was revealed: in the long-term follow-up, chronic stable state, high bilirubin was shown to be a significant protective factor (35% reduction in MACEs risk and 41% decrease in all-cause mortality risk). In contrast, during the in-hospital acute phase (e.g., MI or ACS), high bilirubin was identified as an independent risk marker for short-term adverse events (133% increase in MACEs risk and 179% elevation in CV death risk). Furthermore, the overall unstratified pooled analyses revealed no statistical significance for any outcome (OR close to 1) with extremely high heterogeneity (I^2^ > 78%), fully demonstrating that without distinguishing clinical scenarios, the true prognostic effect of bilirubin would be completely masked. In addition, key effect modifiers were identified, including the composition of MACE endpoints (composite endpoint vs. safety hard endpoint), CHD subtype (mixed CAD vs. isolated AMI), patient age (>65 years), proportion of male patients, and criteria for excluding liver disease, providing significant references for study design in the future. It should be noted that the core objective of this study was not to verify a causal association between bilirubin and cardiovascular outcomes, but rather to elucidate the value of bilirubin as a prognosis marker across different clinical scenarios.

### 4.1 Research progress and source tracing of contradiction

Since Schwertner et al. (3) in 1994 and Breimer et al. (8) in 1995 first reported that low serum total bilirubin was an independent risk factor for coronary artery disease (CAD), the association between bilirubin and CHD has remained highly controversial. Early observational studies have confirmed that the strength of the association between bilirubin and CAD is comparable to that of traditional cardiovascular risk factors such as smoking and systolic blood pressure, suggesting its potential value for cardiovascular protection (9). However, Mendelian randomization studies by Stender et al. (10) in 2012 and by Kunutsor et al. (11, 12) in 2015 have found no causal association between genetically high bilirubin and ischemic heart disease or MI, thereby questioning the clinical predictive value of high bilirubin.

This study provides a key explanation for this decades-long controversy. The core reason for conflicting findings in previous studies is that the pathological stage of the disease, clinical subtype, and follow-up duration are not distinguished, leading to cancellation of the bidirectional effects of bilirubin across different scenarios and ultimately exhibiting a non-significant general association. Moreover, the essential differences in the composition of MACE endpoints across different follow-up scenarios were generally overlooked, which is also a core factor contributing to contradictory conclusions between studies. In the in-hospital follow-up subgroup, MACE endpoints naturally did not include effectiveness-related soft endpoints such as rehospitalization and repeat revascularization, but were mostly composed of acute-phase safety hard endpoints. The definitions of events within this subgroup were highly homogeneous, resulting in very low heterogeneity (I^2^ = 23.6%). In contrast, in the long-term follow-up subgroup, MACE endpoints extensively included soft endpoints related to chronic progression. The differences in event composition were the core reason for the high heterogeneity within this subgroup and the complete reversal of effect direction between the two subgroups. The pathophysiological basis of the two types of endpoints and the clinical significance of bilirubin are fully opposite. Previous studies that did not perform standardized stratification of MACE endpoints and pooled events with opposite pathological meanings inevitably led to cancellation of effect sizes and ultimately contradictory research conclusions.

Previous meta-analyses have already found that the prognostic effect of bilirubin differs significantly between non-MI and MI types of CHD and between short-term and long-term follow-up (13, 14). This study validated and extended these findings in the special population of patients after PCI, who undergo vascular intervention and ischemia-reperfusion injury, and clarified the essential differences in the context-dependent bilirubin effect in this population compared with the general CAD population. Notably, the core objective of this study was not to verify the causal association between bilirubin and cardiovascular outcomes, but rather to clarify the value of bilirubin as a prognostic marker across different clinical scenarios. This differs essentially from the ‘causality verification’ perspective of Mendelian randomization studies. The core logic that ‘the effect attribute is determined by the pathological state’ is supported by basic research on the anti-atherosclerotic effect of bilirubin during the chronic stable phase (15) and perfectly reconciles the discrepancies in conclusions between previous observational studies and Mendelian randomization studies.

### 4.2 Pathophysiological mechanisms of bidirectional prognostic effect

The bidirectional effect of bilirubin observed in this study does not reflect a reversal of its own biological action, but rather a fundamental difference in the pathophysiological meaning of an elevated bilirubin level under different disease states. The specific mechanisms can be explained in two dimensions.

#### 4.2.1 Cardiovascular protection mechanism in a chronic stable state

In the chronic stable phase of non-MI CAD and after elective PCI, bilirubin, as the end product of heme degradation catalyzed by heme oxygenase-1 (HO-1), exerts its protective effects through potent antioxidant, anti-inflammatory, and anti-atherosclerotic activities. In the 1980s, Stocker et al. (16) first revealed that bilirubin is a physiologically important endogenous antioxidant, with lipid antioxidant activity even superior to that of *α*-tocopherol, and its effect is further enhanced under hypoxic conditions. Subsequent studies have further clarified its core protective pathways. First, bilirubin directly scavenges superoxide anions while inhibiting NAD(P)H oxidase activity to block superoxide generation. Through these dual pathways, bilirubin increases the bioavailability of nitric oxide (NO) and improves vascular endothelial function (17, 18). Second, bilirubin mediates the anti-atherosclerotic effect of HO-1 by inhibiting low-density lipoprotein oxidation and reducing vascular endothelial activation, thereby delaying vascular remodeling and restenosis after PCI (19, 20). Third, bilirubin significantly downregulates pro-inflammatory cytokine levels such as tumor necrosis factor-α (TNF-α) and interleukin-1β (IL-1β) and inhibits cellular senescence (21). This also accounts for the significant protective effect of high bilirubin among older individuals (>65 years) in this study, namely, due to the age-related increase in oxidative stress and inflammatory response, the antioxidant and anti-inflammatory effects of bilirubin become more prominent.

#### 4.2.2 Risk marker mechanism in an acute stress state

During the in-hospital acute phase after MI and PCI, an increase in bilirubin is a stress compensatory response of the body to acute myocardial ischemia/reperfusion injury, rather than a causative factor for adverse events. When acute myocardial necrosis occurs in patients with ACS or MI, oxidative stress and the inflammatory response are acutely activated, the HO-1 pathway is upregulated in response to stress, and bilirubin, as a downstream metabolite, increases compensatorily. This also accounts for the extremely low heterogeneity (I^2^ = 23.6%) observed in the in-hospital subgroup in this study. All patients in this subgroup were homogeneous ACS patients in the acute phase, without admixture of patients with chronic stable CHD, resulting in a highly consistent direction of effect and favorable robustness of the results. In short, elevated bilirubin in the acute phase is a ‘consequence of disease severity’ rather than a ‘cause of poor prognosis’, which is fundamentally different from the nature of bilirubin as a reflection of baseline antioxidant capacity in the chronic stable phase.

### 4.3 Clinical translation and practical implications

The core clinical implication of this study is that bilirubin level among individuals with CHD after PCI should not be simply judged as ‘the higher, the better’ or ‘the higher, the worse’. Instead, differential interpretation must be performed based on the clinical subtype, pathological state, and follow-up duration of affected patients. For chronic stable patients with non-MI CAD undergoing elective PCI, a stably elevated bilirubin level during follow-up suggests a reduced risk of MACEs and all-cause mortality and can be used as a complementary marker for a favorable prognosis. This allows for appropriately extended follow-up intervals among low-risk patients during risk stratification. For patients with ACS or MI, an elevated bilirubin level during hospitalization is a high-risk signal, indicating severe myocardial ischemic injury. Intensified in-hospital monitoring and early post-discharge follow-up are needed, and non-cardiovascular causes such as hepatic ischemia or liver injury should be ruled out. For older patients aged >65 years after PCI, the protective effect of bilirubin is more pronounced. Bilirubin can be incorporated into the long-term prognosis assessment system for older patients, with priority given to complete cycle dynamic monitoring combining data on ‘in-hospital and follow-up’ to avoid misjudgment based solely on in-hospital data.

Furthermore, bilirubin testing is low-cost and highly accessible, and may serve as a supplementary indicator for risk stratification of patients after PCI in primary medical institutions. It should be emphasized that the above clinical implications are exploratory and are intended only as a supplementary reference for risk stratification. The implications cannot be used as the sole basis for clinical treatment decisions, nor can they be inferred to indicate a causal association between bilirubin and cardiovascular outcomes.

However, given that this study is exploratory in nature and that substantial heterogeneity exists in the study data, these clinical implications should only be used as a hypothesis reference for subsequent research rather than being directly applied to alter clinical practice.

### 4.4 Limitations

The findings in this study should be interpreted with caution due to the following limitations. First, inconsistency in the composition of MACE endpoints was the primary source of the extremely high heterogeneity in this study. Among the included studies, some used only hard endpoints (e.g., all-cause mortality, MI, stroke, or in-stent thrombosis), while others included soft endpoints (e.g., revascularization or rehospitalization) that are greatly influenced by clinical decision-making. The subjective nature of the judgment criteria for soft endpoints, combined with significant differences in medical resources and treatment strategies across regions, not only reduced comparability between studies but may also have introduced systematic bias into the pooled effect sizes. Subgroup analyses revealed that the results for safety MACE (hard endpoints) were more stable and reliable, more effectively reflecting the true impact of bilirubin on the prognosis of cardiovascular events, whereas the results for composite MACE exhibited higher heterogeneity and limited clinical reference value. Subgroup analyses have identified follow-up duration and CHD subtype as other core sources of heterogeneity. However, due to factors such as the wide range of cutoff values for bilirubin grouping (3.4–22 μmol/L), inconsistent definitions of MACEs, and inconsistent measurement methods and timing, substantial residual heterogeneity remained unexplained. The cutoff values for bilirubin exposure varied greatly across the included studies, lacking uniform judgment criteria, which weakened the cross-study comparability. Moreover, the dichotomous classification approach has inherent limitations and fails to fully capture the true relationship between variables. In this study, the median of the cutoff values from the included studies (10.26 μmol/L) was used as the grouping standard. This value is within the normal physiological range and fails to reflect the prognostic significance of clinical hyperbilirubinemia. Because the cutoff values varied greatly across studies and a unified clinical standard was lacking, the findings in this study are only applicable to the analysis of differences in bilirubin levels within the normal range and cannot be directly generalized to patients with clinical hyperbilirubinemia. The dose-response analysis was based on a small number of included studies with insufficient statistical power. The negative result merely indicated that no significant statistical association was detected, and the risk of false negativity existed. Therefore, this result cannot be used as evidence for the absence of a true dose-response relationship, which This significantly limited the generalizability of the overall pooled effect sizes. Second, classification bias existed in the grouping of CHD subtypes. Detailed subgroup analyses by acute or chronic clinical status and CHD subtype could not be performed. Among the seventeen included studies, seven reported only STEMI, one only AMI, one only NSTEMI, one only ACS, and the remaining seven included mixed CHD cohorts without stratified data. The number of studies for most CHD subtypes was ≤2, far below the statistical power threshold for meta-analysis, and forced stratification would lead to significant result bias. This was a significant source of residual heterogeneity in this study. Limited by the quality of data reported in the original studies, the prespecified mixed CHD subgroup could not be finely disaggregated into CHD subtypes. This subgroup simultaneously contained patients with both chronic stable and acute ischemic stress states, which not only diluted the true association between bilirubin and prognostic outcomes but also increased within-subgroup heterogeneity and reduced the reliability of subgroup conclusions. Third, extremely high residual heterogeneity due to inconsistent definitions of liver disease. Extreme differences existed across the included studies in the definition and exclusion criteria for liver disease, which was the most important source of the extremely high residual heterogeneity in this study. This heterogeneity severely confounded the true prognostic meaning of bilirubin, leading to significant instability in the pooled effect sizes. Even after subgroup analyses and sensitivity analyses, this bias could not be fully eliminated. Fourth, a significant population structure bias existed. Among the 17 included studies, 15 were from East Asia (including 13 from mainland China), and only 2 were from Europe or the Americas. Polymorphisms in bilirubin metabolism-related genes (e.g., Gilbert syndrome associated with the UGT1A1 gene) vary across ethnic groups, which may lead to ethnic heterogeneity in the strength of the association between bilirubin and the prognosis of cardiovascular events. Therefore, the results from this study cannot be directly generalized to all global populations. Fifth, significant limitations existed in the adjustment for confounding factors. Because individual participant data could not be obtained, uniform multivariable adjustment could not be performed across all studies, and significant residual confounding might have existed. Moreover, the number of studies in some key subgroups was small, resulting in insufficient statistical power, and selection and information biases possibly existed in the included retrospective studies. Sixth, methodological and confounding control limitations existed. The included studies had inconsistent adjustment for confounding factors, and residual confounding may have been present. Only total bilirubin was analyzed, and the independent prognostic values of direct and indirect bilirubin were not distinguished. The number of studies included in some key subgroups was small, resulting in insufficient statistical power. Potential selection and information biases also existed in the included retrospective studies. Seventh, no significant linear or nonlinear dose-response association between total bilirubin and MACEs risk after PCI was found in this dose-response meta-analysis, which may be related to the small number of included studies and insufficient statistical power. Eighth, the OR was used as the effect measure in this study. OR only reflects the between-group association of event incidence, ignoring differences in follow-up duration, and does not capture the dynamic changes in temporal prognosis risk. Therefore, the results from this study are only exploratory association analyses and cannot be equated with temporal prognostic risk conclusions. For prognosis meta-analyses in the future, it is recommended that the HR be used as the preferred effect measure to more accurately reflect the temporal impact of bilirubin on long-term prognosis. Ninth, the bidirectional effect findings in this study are highly dependent on subgroup classification. Affected by factors such as study heterogeneity, population characteristics, and criteria for outcome judgment, this phenomenon is only an associative finding and cannot yet be equated with a definitive objective law. Tenth, this study is a meta-analysis based on aggregated data and therefore is influenced by the limitation of ‘ecological fallacy’: the associations observed at the group level cannot be directly extrapolated to individual clinical rules. Eleventh, multiple subgroup analyses were performed without adjustment for the significance level, resulting in ‘multiplicity bias’ and a risk of false-positive findings in the subgroup results.

In summary, the extremely high between-study heterogeneity suggests that simple pooling of effect sizes is influenced by clear statistical limitations. The results from this study should be interpreted only as trend associations rather than definitive quantitative conclusions. Residual heterogeneity mainly originated from baseline disease severity, timing of bilirubin measurement, differences in MACE definitions, and differences related to geography or ethnicity. Accordingly, a conservative and cautious interpretation was adopted for all results in this study.

### 4.5 Future directions

This bidirectional trend represents an exploratory finding. Based on the findings and limitations of this study, research in the future should focus on the following aspects. First, a refined distinction between different pathological subtypes and disease stages of CHD should be drawn to clarify the dynamic changes and differential prognostic value of bilirubin at different phases of the disease course. Second, methodological standards should be unified, including standardization of bilirubin measurement, cutoff value definitions, and MACE endpoint settings, to reduce between-study heterogeneity. Third, the mechanisms of action should be further explored, distinguishing the independent prognostic values of different bilirubin components and integrating genetic polymorphism analyses to clarify the causal association between bilirubin and cardiovascular outcomes. Fourth, prospective cohort studies with multi-regional, multi-ethnic, and large-scale designs are warranted. Fifth, when prospective cohort studies are performed in the future, it is recommended that the collection and adjustment of key confounding factors be standardized, especially those directly related to bilirubin metabolism such as liver disease, inflammation, and hemolysis, and that multivariable regression models be prioritized for adequate adjustment to obtain more reliable causal evidence.

## 5 Conclusion

This study does not confirm that serum total bilirubin has a robust, independent prognostic value for patients with CHD after PCI. Instead, it reveals a context-dependent bidirectional association: in relatively stable clinical states (e.g., long-term follow-up, chronic stable phase), higher bilirubin levels are associated with a lower risk of adverse events; whereas during the in-hospital acute phase (e.g., myocardial infarction or acute coronary syndrome), higher bilirubin levels are associated with a higher risk of adverse events. However, the extremely high between-study heterogeneity (I^2^ > 80% for most outcomes), inconsistent definitions and exclusion criteria for liver disease across studies, and the negative dose-response analysis (likely underpowered) preclude any definitive conclusion. The overall unstratified pooled estimates were also not statistically significant, further indicating that the apparent associations are highly conditional and not generalizable.

Therefore, the findings of this study are exploratory and hypothesis-generating. They offer a possible explanation for the previously observed “bilirubin-CHD paradox” but do not support the use of total bilirubin as a generalizable prognostic biomarker in clinical practice. Future prospective studies with strict standardization of liver disease definitions, MACE endpoint composition, timing of bilirubin measurement, and adequate adjustment for confounders are needed to validate these conditional associations.

## Data Availability

The original contributions presented in the study are included in the article/Supplementary Material, further inquiries can be directed to the corresponding author.

## Supplementary material

Supplementary Table 1 Search strategy.

Supplementary Table 2 PRISMA 2020 checklist for the meta-analysis.

Supplementary Table 3 Definition and Exclusion Criteria of Liver Disease in Included Studies.

Supplementary Table 4 Univariable Meta-Regression of MACEs.

Supplementary Table 5 Univariable Meta-Regression of CV Death.

Supplementary Table 6 Univariable Meta-Regression of All-Cause Mortality.

Supplementary Figure 1 Overall unstratified forest plots for the association between serum total bilirubin and clinical outcomes after PCI.

Supplementary Figure 2 Dose–response meta-analysis of total bilirubin and long-term follow-up MACEs.

Supplementary Figure 3 Leave-one-out sensitivity analyses for the association between serum total bilirubin and clinical outcomes within the long-term follow-up subgroup.
