# Supplementary material for: Prognostic value of total bilirubin level for patients suffering from coronary heart disease undergoing percutaneous coronary intervention: a meta-analysis
Source: Front Cardiovasc Med. 2026 Jul 30;13:1783537. doi: 10.3389/fcvm.2026.1783537 (PMC13467939; doi:10.3389/fcvm.2026.1783537)
Supplement: Supplementary file 1 [file Datasheet1.docx]

**Supplementary Material**

**Supplementary Table 1** PRISMA 2020 Checklist for the Meta-Analysis

| **Section** | **Item No.** | **PRISMA 2020 Checklist Item** | **Completed (Yes/No/NA)** | **Location in Manuscript** | **Notes** |
| --- | --- | --- | --- | --- | --- |
| Title | 1 | Identify the report as a systematic review, meta-analysis, or both. | Yes | Title Page | Explicitly labeled as a meta-analysis in the title |
| Abstract | 2 | Provide a structured summary including: background, objectives, data sources, eligibility criteria, study appraisal, synthesis methods, results, limitations, conclusions, registration number. | Yes | Abstract | Structured abstract covers all required elements; PROSPERO registration noted |
| Introduction | 3 | Describe the rationale for the review in the context of existing evidence. | Yes | 1. Introduction | Clarified the "bilirubin-CHD paradox" and research gap in post-PCI population |
|  | 4 | State explicit research questions using PICOS. | Yes | Abstract + 2.2 | Clear PICOS-based objective for prognostic value of total bilirubin |
| Methods | 5 | Indicate review protocol, registration, and access. | Yes | 2. Methods (opening) | Registered in PROSPERO; registration number provided |
|  | 6 | Specify eligibility criteria (PICOS, study characteristics, publication restrictions). | Yes | 2.2 | Full PICOS criteria; English observational studies only |
|  | 7 | Describe all information sources and search dates. | Yes | 2.1 | 4 databases (PubMed, Cochrane Library, Embase, Web of Science) to May 22, 2026; manual reference search |
|  | 8 | Present full, reproducible electronic search strategy for at least one database. | Yes | Supplementary Table S1 | Complete search strategy provided for all databases |
|  | 9 | Describe study selection process (screening, eligibility, inclusion). | Yes | 2.3 + 3. Results | Dual independent screening; PRISMA flow diagram (Figure 1) |
|  | 10 | Describe data extraction process (duplicate extraction, author contact). | Yes | 2.3 | Dual independent extraction; disagreements resolved by third reviewer |
|  | 11 | List all data items extracted (PICOS, outcomes, covariates). | Yes | 2.3 + Table 1 | Extracted study characteristics, bilirubin metrics, follow-up, MACE definitions, effect sizes |
|  | 12 | Describe risk of bias assessment for individual studies. |  | 2.4 + Tables 2 & 3 | Newcastle‑Ottawa Scale (NOS) used for cohort and case‑control studies; dual assessment. |
|  | 13 | Specify summary effect measures for each outcome. | Yes | 2.5 | OR (95% CI) for binary outcomes; SMD (95% CI) for continuous variables. |
|  | 14 | Describe synthesis methods: meta-analysis, heterogeneity, subgroup, sensitivity, meta-regression. | Yes | 2.5 + 3.3 | Random‑effects model; I² and Q test; prespecified subgroup analyses (by follow‑up setting, age, sex, etc.); leave‑one‑out sensitivity; univariate meta‑regression; dose‑response analysis. |
|  | 15 | Describe methods to assess risk of bias across studies (publication bias). | Yes | 2.5 + 3.3.5 | Begg’s test, Egger’s test, and funnel plot for MACEs. |
| Results | 16 | Report study selection flow (numbers screened, eligible, included; reasons for exclusion). | Yes | 3. Results (first paragraph) + Figure 1 | 720 records identified, 131 duplicates removed, 27 full‑text assessed, 17 studies finally included. |
|  | 17 | Present characteristics of included studies. | Yes | 3.1 + Table 1 | 17 studies (27,580 patients); detailed characteristics (country, CHD subtype, follow‑up duration, bilirubin measurement timing, cutoff values, MACE definitions, liver disease exclusion). |
|  | 18 | Present risk of bias for included studies. | Yes | 3.2 + Tables 2 & 3 | All cohort studies scored 7–9 stars; all case‑control studies scored 7 stars (high quality). |
|  | 19 | Present individual study results for all outcomes. | Yes | 3.3.1 (Table 4) + Supplementary Figures 1A–D, 2, 3A–C | Individual study effect sizes are presented in forest plots (overall and stratified). |
|  | 20 | Present synthesis results (pooled effect, CI, heterogeneity). | Yes | 3.3.1 (Table 4) | Pooled ORs, 95% CIs, I² values, and P for heterogeneity are reported for all outcomes in each subgroup. |
|  | 21 | Report results of risk of bias across studies (publication bias). | Yes | 3.3.5 (Sensitivity analysis and publication bias) + Figure 3 | Begg’s test and Egger’s test P values reported for MACEs, CV death, and all‑cause mortality; no significant publication bias. Funnel plot for MACEs (Figure 3). |
|  | 22 | Report results of additional analyses (subgroup, sensitivity, meta-regression, dose-response). | Yes | 3.3.2, 3.3.3, 3.3.4, 3.3.5, 3.3.6 + Tables 5,6,7 and Supplementary Tables 4–6 | Additional analyses include: further subgroup analyses within long‑term follow‑up (Tables 5–7), dose‑response (Supplementary Figure 2), leave‑one‑out sensitivity (Supplementary Figure 3A–C), and univariate meta‑regression (Supplementary Tables 4–6). |
| Discussion | 23 | Summarize main findings and strength of evidence. | Yes | 4. Discussion + 4.3 | Context‑dependent bidirectional prognostic effect of bilirubin is summarised; clinical implications discussed. |
|  | 24 | Discuss limitations of included studies and review. | Yes | 4.4 | Limitations include heterogeneity in MACE endpoints, CHD subtype classification, liver disease definitions, population bias, and methodological issues. |
|  | 25 | Provide general interpretation and implications for future research. | Yes | 4.5 + 5. Conclusion | Future directions proposed: refined pathological subtyping, unified methodological standards, mechanism exploration, and large‑scale prospective cohorts. |
| Other | 26 | Describe funding sources and funder role. | Yes | Funding section | Funding sources declared; no role of funders in study design, analysis, or publication. |
|  | 27 | Declare competing interests. | Yes | Competing interests | Authors declare no competing interests. |

**Supplementary Table 2** Search Strategy

Embase

| No. | Query | Results | Date |
| --- | --- | --- | --- |
| #7 | #3 AND #6 | 542 | 22-May-26 |
| #6 | #4 OR #5 | 173029 | 22-May-26 |
| #5 | '1, 3, 6, 7 tetramethyl 4, 5 dicarboxyethyl 2, 8 divinyl b 13 dihydrobilenone':ti,ab,kw OR 'bilirubin':ti,ab,kw OR 'bilirubinate':ti,ab,kw OR 'bilirubine':ti,ab,kw OR 'bilirubinoid':ti,ab,kw OR 'haematoidin':ti,ab,kw OR 'hematoidin':ti,ab,kw OR 'mesobilirubin':ti,ab,kw | 94837 | 22-May-26 |
| #4 | 'bilirubin'/exp | 153080 | 22-May-26 |
| #3 | #1 OR #2 | 192657 | 22-May-26 |
| #2 | 'percutaneous coronary intervention*':ti,ab,kw OR 'percutaneous coronary revascularization*':ti,ab,kw OR 'pci':ti,ab,kw | 155615 | 22-May-26 |
| #1 | 'percutaneous coronary intervention'/exp | 129866 | 22-May-26 |

Cochrane

| ID | Search | Results |
| --- | --- | --- |
| #1 | MeSH descriptor: [Percutaneous Coronary Intervention] explode all trees | 9090 |
| #2 | (‘percutaneous coronary intervention*’ OR ‘Percutaneous Coronary Revascularization*’ OR ‘PCI’):ti,ab,kw | 18776 |
| #3 | #1 OR #2 | 20836 |
| #4 | MeSH descriptor: [Bilirubin] explode all trees | 1084 |
| #5 | (‘1, 3, 6, 7 tetramethyl 4, 5 dicarboxyethyl 2, 8 divinyl b 13 dihydrobilenone’ OR ‘bilirubin’ OR ‘bilirubinate’ OR ‘bilirubine’ OR ‘bilirubinoid’ OR ‘haematoidin’ OR ‘hematoidin’ OR ‘mesobilirubin’):ti,ab,kw | 11347 |
| #6 | #4 OR #5 | 11347 |
| #7 | #3 AND #6 | 19 |

Pubmed

| Search number | Query | Search Details | Results | Time |
| --- | --- | --- | --- | --- |
| 7 | #3 AND #6 | ("percutaneous coronary intervention"[MeSH Terms] OR ("percutaneous coronary intervention*"[Title/Abstract] OR "percutaneous coronary revascularization*"[Title/Abstract] OR "PCI"[Title/Abstract])) AND ("bilirubin"[MeSH Terms] OR ("bilirubin"[Title/Abstract] OR "bilirubinate"[Title/Abstract] OR "bilirubine"[Title/Abstract] OR "bilirubinoid"[Title/Abstract] OR "haematoidin"[Title/Abstract] OR "hematoidin"[Title/Abstract] OR "mesobilirubin"[Title/Abstract])) | 66 | 10:41:31 |
| 6 | #4 OR #5 | "bilirubin"[MeSH Terms] OR "bilirubin"[Title/Abstract] OR "bilirubinate"[Title/Abstract] OR "bilirubine"[Title/Abstract] OR "bilirubinoid"[Title/Abstract] OR "haematoidin"[Title/Abstract] OR "hematoidin"[Title/Abstract] OR "mesobilirubin"[Title/Abstract] | 60,979 | 10:41:22 |
| 5 | "1, 3, 6, 7 tetramethyl 4, 5 dicarboxyethyl 2, 8 divinyl b 13 dihydrobilenone"[Title/Abstract] OR "bilirubin"[Title/Abstract] OR "bilirubinate"[Title/Abstract] OR "bilirubine"[Title/Abstract] OR "bilirubinoid"[Title/Abstract] OR "haematoidin"[Title/Abstract] OR "hematoidin"[Title/Abstract] OR "mesobilirubin"[Title/Abstract] | "bilirubin"[Title/Abstract] OR "bilirubinate"[Title/Abstract] OR "bilirubine"[Title/Abstract] OR "bilirubinoid"[Title/Abstract] OR "haematoidin"[Title/Abstract] OR "hematoidin"[Title/Abstract] OR "mesobilirubin"[Title/Abstract] | 51,589 | 10:41:13 |
| 4 | Bilirubin[MeSH Terms] | "bilirubin"[MeSH Terms] | 27,857 | 10:41:01 |
| 3 | #1 OR #2 | "percutaneous coronary intervention"[MeSH Terms] OR "percutaneous coronary intervention*"[Title/Abstract] OR "percutaneous coronary revascularization*"[Title/Abstract] OR "PCI"[Title/Abstract] | 100,343 | 10:40:48 |
| 2 | ((percutaneous coronary intervention*[Title/Abstract]) OR (Percutaneous Coronary Revascularization*[Title/Abstract])) OR (PCI[Title/Abstract]) | "percutaneous coronary intervention*"[Title/Abstract] OR "percutaneous coronary revascularization*"[Title/Abstract] OR "PCI"[Title/Abstract] | 67,396 | 10:40:35 |
| 1 | Percutaneous Coronary Intervention[MeSH Terms] | "percutaneous coronary intervention"[MeSH Terms] | 71,352 | 10:40:16 |

Wos

| Entitlements | # | Search Query | Database | Results | Date Run |
| --- | --- | --- | --- | --- | --- |
| - WOS.IC: 1993 to 2026 - WOS.CCR: 1985 to 2026 - WOS.SCI: 1999 to 2026 | 2 | TS=(((percutaneous coronary intervention*) OR (Percutaneous Coronary Revascularization*) OR (PCI)) ) | Web of Science Core Collection | 104555 | Fri May 22 2026 22:24:14 GMT+0800 (中国标准时间) |
| - WOS.IC: 1993 to 2026 - WOS.CCR: 1985 to 2026 - WOS.SCI: 1999 to 2026 | 3 | TS=(((1, 3, 6, 7 tetramethyl 4, 5 dicarboxyethyl 2, 8 divinyl b 13 dihydrobilenone) OR (bilirubin) OR (bilirubinate) OR (bilirubine) OR (bilirubinoid) OR (haematoidin) OR (hematoidin) OR (mesobilirubin)) ) | Web of Science Core Collection | 48614 | Fri May 22 2026 22:25:03 GMT+0800 (中国标准时间) |
| - WOS.IC: 1993 to 2026 - WOS.CCR: 1985 to 2026 - WOS.SCI: 1999 to 2026 | 4 | #3 AND #2 | Web of Science Core Collection | 93 | Fri May 22 2026 22:25:22 GMT+0800 (中国标准时间) |

**Supplementary Table 3** Definitions and Exclusion Criteria for Liver Disease in the Included Studies.

| First Author | Publication Year | Exclusion of Prior Liver Disease | Definition / Criteria | Biochemical Confirmation (Yes/No) |
| --- | --- | --- | --- | --- |
| Zhang | 2017 | Yes | Patients with a history of kidney and hepatic diseases | No |
| Huang | 2012 | Yes | Patients with a history or clinical evidence of renal failure in chronic peritoneal or hemodialytic treatment, alcoholism or chronic liver disease were also excluded. | No |
| Fang | 2022 | Yes | combined with severe liver and kidney dysfunction. | No |
| Li | 2022 | Yes | Acute or chronic liver injury  Acute liver injury was screened with an acute elevation of transaminases. Chronic liver injury was screened mainly by chronic elevation of transaminases with the case history, such as viral hepatitis, fatty liver disease, alcoholic hepatitis, autoimmune liver disease, biliopancreatic disease, drug-induced liver injury, liver cancer, liver cirrhosis and so on. | Yes |
| Gao | 2019 | Yes | patients with serum TB at admission >2.0 mg/dL | Yes |
| Chung | 2016 | Yes | known liver disease, end-stage renal disease | No |
| Acet | 2014 | Yes | severe liver disease | No |
| Tuxun | 2020 | Yes | severe liver and kidney diseases | No |
| Ying | 2023 | Yes | **liver and kidney dysfunction** | No |
| Yao | 2015 | Yes | chronic kidney disease, chronic hepatic disease, | No |
| Gul | 2013 | Yes | known liver, hematologic | No |
| Zhao | 2020 | No |  |  |
| Wei | 2014 | No |  |  |
| Zhang | 2014 | Yes | severe hepatic injury (alanine aminotransferase (ALT)>80 U/L) | Yes |
| Yang | 2022 | Yes | hepatic or renal dysfunction that might  affect serum TBIL | No |
| Kim | 2015 | No |  |  |
| Yu | 2026 | Yes | severe hepatic or kidney dysfunction | No |

**Supplementary Table 4** Univariable Meta-Regression of MACEs

| Covariate | Regression Coefficient (Coef.) | Standard Error (Std. Err.) | t-value | P-value | 95% Confidence Interval (CI) for Coefficient | Residual Heterogeneity (I²_res) | Adjusted R² (Adj R²) |
| --- | --- | --- | --- | --- | --- | --- | --- |
| Mean age | −0.059 | 0.070 | −0.84 | 0.421 | −0.215 to 0.097 | 85.85% | −3.65% |
| CHD subtype | −0.586 | 0.478 | −1.23 | 0.248 | −1.652 to 0.479 | 87.69% | 1.06% |
| MACE endpoint definition | −0.567 | 0.510 | −1.11 | 0.292 | −1.703 to 0.569 | 85.37% | 5.54% |
| Total bilirubin cutoff value | −0.144 | 0.522 | −0.28 | 0.789 | −1.306 to 1.019 | 87.67% | -14.13% |
| Exclusion of prior liver disease | 1.102 | 0.633 | 1.74 | 0.112 | −0.309 to 2.513 | 85.55% | 20.80% |

**Supplementary Table 5** Univariable Meta-Regression of CV Death

| **Covariate** | **Regression Coefficient (Coef.)** | **Standard Error (Std. Err.)** | **t-value** | **P-value** | **95% Confidence Interval (CI) for Coefficient** | **Residual Heterogeneity (I²_res)** | **Adjusted R² (Adj R²)** |
| --- | --- | --- | --- | --- | --- | --- | --- |
| Mean age | -0.095 | 0.059 | -1.61 | 0.152 | -0.235 to 0.045 | 68.83% | 41.96% |
| CHD subtype | -0.215 | 0.613 | -0.35 | 0.736 | -1.665 to 1.234 | 81.06% | -30.07% |
| Total bilirubin cutoff value | -0.501 | 0.590 | -0.85 | 0.424 | -1.897 to 0.895 | 81.57% | -14.36% |
| Exclusion of prior liver disease | 1.520 | 0.706 | 2.15 | 0.068 | -0.149 to 3.189 | 76.82% | 32.05% |

**Supplementary Table 6** Univariable Meta-Regression of All-Cause Mortality

| **Covariate** | **Regression Coefficient (Coef.)** | **Standard Error (Std. Err.)** | **t-value** | **P-value** | **95% Confidence Interval (CI) for Coefficient** | **Residual Heterogeneity (I²_res)** | **Adjusted R² (Adj R²)** |
| --- | --- | --- | --- | --- | --- | --- | --- |
| Mean age | -0.034 | 0.072 | -0.46 | 0.663 | -0.220 to 0.153 | 56.58% | -106.32% |
| CHD subtype | -0.425 | 0.552 | -0.77 | 0.476 | -1.845 to 0.994 | 58.55% | -96.38% |
| Total bilirubin cutoff value | -0.066 | 0.574 | -0.11 | 0.914 | -1.541 to 1.410 | 59.51% | -135.63% |
| Exclusion of prior liver disease | 0.626 | 0.936 | 0.67 | 0.533 | -1.779 to 3.032 | 58.23% | -23.65% |

**Supplementary Figure 1** Overall unstratified forest plots for the association between serum total bilirubin and clinical outcomes after PCI; A Major adverse cardiovascular events (MACEs); B Cardiovascular death; C All‑cause mortality; D Difference in serum total bilirubin level between patients with and without MACEs (standardized mean difference, SMD)

**Supplementary Figure 2** Dose–response meta-analysis of total bilirubin and long-term follow-up MACEs

**Supplementary Figure 3** Leave‑one‑out sensitivity analyses for the association between serum total bilirubin and clinical outcomes within the long‑term follow‑up subgroup; A Major adverse cardiovascular events (MACEs); B Cardiovascular death; C All‑cause mortality
